# Detection of *Salmonella* Typhi nucleic acid by RT-PCR and anti-HlyE, -CdtB, -PilL, and -Vi IgM by ELISA at sites in Ghana, Madagascar and Ethiopia

**DOI:** 10.1186/s12879-022-07726-3

**Published:** 2022-10-02

**Authors:** Ursula Panzner, Ondari Daniel Mogeni, Yaw Adu-Sarkodie, Trevor Toy, Hyon Jin Jeon, Gi Deok Pak, Se Eun Park, Yeetey Enuameh, Ellis Owusu-Dabo, Trinh Van Tan, Abraham Aseffa, Mekonnen Teferi, Biruk Yeshitela, Stephen Baker, Raphael Rakotozandrindrainy, Florian Marks

**Affiliations:** 1 International Vaccine Institute, SNU Research Park, 1 Gwanak-ro, Gwanak-gu, Seoul, 08826 South Korea; 2 Kumasi Centre for Collaborative Research in Tropical Medicine, Kwame Nkrumah University of Science and Technology, Kumasi, Ghana; 3 School of Medicine and Dentistry, Kwame Nkrumah University of Science and Technology (KNUST), Kumasi, Ghana; 4 Cambridge Institute of Therapeutic Immunology and Infectious Disease, University of Cambridge School of Clinical Medicine, Cambridge Biomedical Campus, Cambridge, CB2 0AW UK; 5 Kintampo Health Research Center (KHRC), Kintampo, Ghana; 6 School of Public Health, Kwame Nkrumah University of Science and Technology, Kumasi, Ghana; 7 Oxford University Clinical Research Unit, Ho Chi Minh City, Vietnam; 8 Armauer Hansen Research Institute, Ministry of Health, Addis Ababa, Ethiopia; 9 Madagascar Institute for Vaccine Research, University of Antananarivo, Antananarivo, Madagascar; 10 Heidelberg Institute of Global Health, University of Heidelberg, Heidelberg, Germany; 11 Swiss Tropical and Public Health Institute, Basel/Allschwil, Switzerland; 12 University of Basel, Basel, Switzerland

**Keywords:** ELISA, HlyE-/CdtB-/PilL-/Vi-antigen, *Salmonella*, Febrile illness, Surveillance

## Abstract

**Background:**

We aimed to assess the prevalence of *Salmonella* Typhi through DNA and IgM-antibody detection methods as a prelude to extended surveillance activities at sites in Ghana, Madagascar, and Ethiopia.

**Methods:**

We performed species-specific real-time polymerase reaction (RT-PCR) to identify bacterial nucleic acid, and enzyme-linked immunosorbent assay (ELISA) for detecting HlyE/STY1498-, CdtB/STY1886-, pilL/STY4539- and Vi-antigens in blood and biopsy specimens of febrile and non-febrile subjects. We generated antigen-specific ELISA proxy cut-offs by change-point analyses, and utilized cumulative sum as detection method coupled with 1000 repetitive bootstrap analyses. We computed prevalence rates in addition to odds ratios to assess correlations between ELISA outcomes and participant characteristics.

**Results:**

Definitive positive RT-PCR results were obtained from samples of febrile subjects originating from Adama Zuria/Ethiopia (1.9%, 2/104), Wolayita Sodo/Ethiopia (1.0%, 1/100), Diego/Madagascar (1.0%, 1/100), and Kintampo/Ghana (1.0%, 1/100), and from samples of non-febrile subjects from Wolayita Sodo/Ethiopia (1%, 2/201). While IgM antibodies against all antigens were identified across all sites, prevalence rates were highest at all Ethiopian sites, albeit in non-febrile populations. Significant correlations in febrile subjects aged < 15 years versus ≥ 15 years were detected for Vi (Odds Ratio (OR): 8.00, *p* = 0.034) in Adama Zuria/Ethiopia, STY1498 (OR: 3.21, *p* = 0.008), STY1886 (OR: 2.31, *p* = 0.054) and STY4539 (OR: 2.82, *p* = 0.022) in Diego/Madagascar, and STY1498 (OR: 2.45, *p* = 0.034) in Kintampo/Ghana. We found statistical significance in non-febrile male *versus* female subjects for STY1498 (OR: 1.96, *p* = 0.020) in Adama Zuria/Ethiopia, Vi (OR: 2.84, *p* = 0.048) in Diego/Madagascar, and STY4539 (OR: 0.46, *p* = 0.009) in Kintampo/Ghana.

**Conclusions:**

Findings indicate non-discriminatory stages of acute infections, though with site-specific differences. Immune responses among non-febrile, presumably healthy participants may mask recall and/or reporting bias leading to misclassification, or asymptomatic, subclinical infection signs induced by suppression of inflammatory responses. As most Ethiopian participants were ≥ 15 years of age and not at high-risk, the true *S.* Typhi burden was likely missed. Change-point analyses for generating ELISA proxy cut-offs appeared robust, though misclassification is possible. Our findings provided important information that may be useful to assess sites prior to implementing surveillance for febrile illness including *Salmonella* disease.

**Supplementary Information:**

The online version contains supplementary material available at 10.1186/s12879-022-07726-3.

## Background

Community-acquired febrile diseases remain a major cause of morbidity and mortality across sub-Saharan Africa. The management of these diseases presents critical challenges for healthcare personnel because of their non-specific clinical presentations coupled with limited diagnostic capacities [1, 2]. This frequently results in the unnecessary use of antimicrobial drugs, which promotes emerging bacterial resistance [1, 2]. Human-pathogenic febrile *Salmonella* disease is reported less frequently from Africa compared to Asia largely because its prevalence is assessed via cross-sectional disease investigations at settings scattered across the continent than long-term disease monitoring [3, 4]. Estimates of the World Health Organization (WHO) stated that *S.* Typhi, the etiologic agent of typhoid fever, is responsible for as many as 20 million cases per year worldwide leading to 128,000–161,000 deaths [5]. These findings, along with the devastating impact of recent epidemics due to Ebola virus and coronavirus/COVID-19, both pathogens presenting initially with mild to moderate infectious disease symptoms among individuals without underlying comorbidities, emphasize the importance of public health surveillance. Methods to assess fever etiologies combined with means to consolidate existing and new data points support preparedness and timely responses to disease outbreaks and emergencies [5–8].

To fill the knowledge gap of the burden of community-acquired febrile illness in Africa, and to build on findings from the multi-country, multi-center Typhoid Fever Surveillance in Africa Program (TSAP) conducted during 2010–2014, research on fever and particular on *Salmonella* disease continued under its successor, i.e., the Severe Typhoid Fever in Africa (SETA) program [9–13]. SETA was implemented during 2016–2019 to assess the burden, severity, and long-term sequelae of salmonellosis, including the identification of carriers and costs associated with this illness. Evidence generated by both programs will strengthen the decision-making on the prevention of *Salmonella* disease through vaccination, and will provide insights into considerations of country-specific adaptations for the management of febrile illness.

Although both programs (TSAP and SETA) implemented fever surveillance at numerous settings, new sites covering additional geographic areas were proposed in Ghana, Madagascar and Ethiopia for extended surveillance activities. We, therefore, aimed to assess the presence of *S.* Typhi in these sites via IgM-antibody detection directed against selected antigens by enzyme-linked immunosorbent assay (ELISA), and species-specific nucleic acid amplification by real-time polymerase chain reaction (RT-PCR) in both, blood and surgical biopsy samples, of acute febrile subjects (FSs) and non-febrile subjects (NFSs) serving as unmatched comparator cohorts.

## Methods

### Study sites

The following sites were selected for extended surveillance: Kintampo in Ghana, Diego and Mahajanga in Madagascar, and Addis Ketema Woreda-7/-8 in Addis Ababa, Adama Wenji Zuria, Debre Birhan, Arba Minch and Wolayita Sodo in Ethiopia (Additional file 1: Fig. S1A). The Kintampo site was selected because of its participation in the International Network for the Demographic Evaluation of Populations and Their Health (INDEPTH) network. The Malagasy sites were chosen based on their coastal location, a presumed high disease burden, and following discussions with Malagasy country partners. In Mahajanga, previously published information on typhoid fever also existed [14]. The Ethiopian sites were included in consultation with the Ethiopian Health Management Information System and the Federal Ministry of Health based on contemporary unpublished reports on the prevalence of diarrheal illness. Site boundaries were selected based on administrative units/municipalities within catchment areas of public, primary and secondary/tertiary recruitment healthcare facilities providing general healthcare; demographic data were extracted from most up-to-date census and statistical services, and were projected to 2016 using annual growth rates (Table 1) [15–22].

**Table 1:** Eligibility criteria for febrile and non-febrile subjects in Ghana, Madagascar and Ethiopia including recruitment healthcare facilities, site boundaries and demographic data

Country	Eligibility criteria		Site	Recruitment HCFs	Study/Catchment area	Population (2016)	
Ghana	FSs	Fever history of ≥ 3 days (72 h) OR temperature of ≥ 38.0 °C (tympanic)/ ≥ 37.5 °C (axillary) OR clinically suspected/diagnosed *Salmonella* infection AND malaria positivity (by RDT/microscopy)	Kintampo	Kintampo Municipal Hospital (Kintampo Health Research Centre) and Yezura Hospital	Municipalities Babatokuma, Busuama, Dawadawa No.2, Gulumpe, Kadelso, Kawampe/Kawompe, Kintampo, Kunsu, New Longoro/Mentukwa and Portor within Kintampo North District; municipalities Agyina/Ajina, Amoma, Ampoma, Anyima, Apesika, Chirehin, Jema, Krabonso, Nante, Ntankoro and Pramposo within Kintampo South District	Total	202,525
						Male	102,629
						Female	99,896
						< 15 yrs	85,843
	NFSs	Self-reported absence of fever history OR clinical indication of fever OR an infectious pathology within the past 28 days				≥ 15 yrs	116,683
Madagascar	FSs	Fever history of ≥ 3 days (72 h) OR temperature of ≥ 38.0 °C (tympanic)/ ≥ 37.5 °C (axillary) OR clinically suspected/diagnosed *Salmonella* infection	Diego	CSB II Tanambao, Kabari Hospital, Tanambo Hospital	Diego or Antsiranana City	Total	439,706
						Male	219,413
						Female	220,293
						< 15 yrs	181,599
						≥ 15 yrs	258,108
	NFSs	Self-reported absence of fever history OR clinical indication of fever OR an infectious pathology within the past 28 days	Mahajanga	CSB II Tanambao-Sotema, Centre Hopitalier Universitaire Mahavoky Atsimo, Centre Hospitalier Universitaire Zafisaona Gabriel	Mahajanga or Majunga City	Total	562,300
						Male	280,588
						Female	281,712
						< 15 yrs	232,230
						≥ 15 yrs	330,070
Ethiopia	FSs	Fever history of ≥ 3 days (72 h) OR temperature of ≥ 38.0 °C (tympanic)/ ≥ 37.5 °C (axillary) AND clinically suspected/diagnosed *Salmonella* infection OR unknown causes for febrile illness	Addis Ketema W7	Millennium Health Center, St. Paul Hospital, Ras Desta Hospital	Municipalities 14, 29, 30 and 32 within District 7 of Addis Ababa	Total	23,490
						Male	12,184
						Female	11,943
						< 15 yrs	10,044
						≥ 15 yrs	14,083
			Addis Ketema W8	Addis Raey Health Center, St. Paul Hospital, Ras Desta Hospital	Municipalities 2–6 and 9–12 within District 8 of Addis Ababa	Total	36,227
						Male	18,295
						Female	17,932
						< 15 yrs	15,081
						≥ 15 yrs	21,146
			Debre Birhan	Debre Birhan Health Center, Debre Birhan Referral Hospital	Municipalities 3–4, 6 and Chole within Debre Birhan Town of Northern Shoa Zone within Amhara Region	Total	34,267
						Male	17,305
						Female	16,962
						< 15 yrs	14,266
						≥ 15 yrs	20,002
	NFSs	Self-reported absence of fever history OR clinical indication of fever OR an infectious pathology within the past 28 days	Arba Minch	Arba Minch Health Center, Arba Minch Hospital	Municipalities Woze, Kulifo, Dil Fana, Menahiria, Gurba, Ediget Ber, Mehal Ketema, Wuha Minch within Arba Minch Town of Gamo Gofa Zone within Southern Nations, Nationalities and People’s Region	Total	82,740
						Male	41,783
						Female	40,956
						< 15 yrs	34,444
						≥ 15 yrs	48,295
			Adama Zuria	Wenji Gefersa Health Center, Wenji Kuriftu Health Center, Adama Hospital Medical College	Municipalities Wenji Gefersa, Wenji Kuriftu, Didimtu, Bati Bora, Bati Germama, and Adulala Boku within Adama Zuria District of Eastern Shoa Zone within Oromia Region	Total	18,694
						Male	9440
						Female	9253
						< 15 yrs	7782
						≥ 15 yrs	10,912
			Wolayita Sodo	Sodo Town Health Center, Wolayita Sodo Zone Hospital	Municipalities Gebeya, Dil Betigil, Gido, Gola, Damota, Alem Damota, and Otona Kodo within Sodo District of Wolayita Zone within Southern Nations, Nationalities and People’s Region	Total	67,619
						Male	34,148
						Female	33,471
						< 15 yrs	28,150
						≥ 15 yrs	39,469

HCF = healthcare facility; yrs = years; FS = febrile subject; NFS = non-febrile subject; W7 = Woreda 7; W8 = Woreda 8

### Study enrollment

Before the study initiation, study teams were trained on project and consent procedures according to Standard Operating Procedures including the handling and testing of participant samples. Acute FSs and NFSs of all ages and both sexes that presented at in-patient and out-patient departments and surgical units of recruitment healthcare facilities were assessed for their eligibility by trained study physicians before study inclusion. Inclusion criteria for FSs differed by country because of the assumed high numbers of febrile illness caused by infections of the respiratory tract, the urinary tract, and the gastrointestinal tract in Ethiopia, and high suspicion of malaria co-infections in Ghana. Fever was defined uniformly across all sites as fever history of ≥ 3 days (72 h) OR tympanic body temperature of ≥ 38.0 °C or axillary body temperature of ≥ 37.5 °C. See Table 1 for more details on the eligibility criteria.

Our goal was to recruit a convenience sample of 100 FSs and 200 NFSs per site during a three-month study period, and to ensure that NFSs visiting for routine health check-ups were enrolled simultaneously to FSs. Upon enrollment, data on demographics and specimen collection were recorded for FSs and NFSs. Data on physical examinations, pre-treatment with antimalarials and/or antibiotics, and clinical appraisals performed by trained study physicians were captured for FSs. All febrile subjects received treatment in accordance with country-specific national standards of care.

### Sample handling and testing

Single whole blood samples ≥ 1.0 mL were collected from FSs and NFSs who were < 16 years of age; samples ≥ 2.0 mL were collected from FSs and NFSs who were ≥ 16-years-old. We examined blood from FSs for the presence of malaria parasites using microscopy or rapid diagnostic testing prior to transfer blood into sterile EDTA-collection tubes for subsequent separation into blood cells and plasma by centrifugation. Tissue biopsies were obtained from FSs undergoing surgical procedures due to disease complications such as gastrointestinal perforation. Biopsy specimens of 5–10 mm^3^ were taken, if feasible, without compromising the surrounding tissue and placed into vials containing 500 µl sterile phosphate-buffered saline. All samples were stored at − 80 °C.

*S.* Typhi IgM ELISA antigens detected by protein microarrays performed in previous investigations of acute naturally acquired infection included hemolysin E (HlyE)/STY1498, cytolethal distending toxin subunit B homolog (CdtB)/STY1886, pilL/STY4539, and Vi. Methods for amplification, gene expression and purification of antigen-containing plasmid constructs were described previously [6, 23]; similarly, ELISA testing was done as described earlier [6, 24]. Briefly, 96-well microtiter plates were coated with 7 µg/ml STY4539, STY1886 or STY1498 or 1 µg/ml Vi in 50 mM carbonate-bicarbonate buffer final concentration per well. We used 5% milk solution prepared in phosphate-buffered saline for washing and blocking throughout. Antigen-coated plates were incubated with 100 µl/well of 1:200 diluted participant plasma followed by 100 µl/well of alkaline phosphate-conjugated anti-human IgM serum. Plates were developed using the p-nitrophenyl phosphate substrate. Absorbance of optical density (OD) values was read at 405 nm and 490 nm wavelength by an automated plate reader.

*S.* Typhi RT-PCR was performed as described previously using freshly extracted DNA from blood cells and tissue biopsies according to Qiagen’s instructions for the QIAamp DNA Blood Midi Kit [25, 26]. Amplification reactions of 25 µl volume were prepared that contained 5 mM MgCl_2_, 0.2 mM each deoxynucleotide triphosphate, 1 U Taq DNA polymerase, and 5 µl template DNA. Final reaction concentrations of species-specific primers, i.e., ST-Frt 5′-CGC-GAA-GTC-AGA-GTC-GAC-ATA-G-3′, ST-Rrt 5′-AAG-ACC-TCA-ACG-CCG-ATC-AC-3′, and the probe, i.e., ST-Probe 5′-FAM-CAT-TTG-TTC-TGG-AGC-AGG-CTG-ACG-GTA-MRA-3′, were 0.4 µM and 0.15 µM, respectively. We utilized cycling conditions of 15 min at 95 °C followed by 45 cycles of 30 s at 95 °C, 30 s at 60 °C, and 30 s at 72 °C. The *Escherichia coli* VU1 strain harboring the gB gene from the Phocid herpes virus served as an internal control to monitor the efficacy of DNA extraction and DNA amplification. We set the following diagnostic crossing points: < 35 cycles as definitive positive, ≥ 35 to < 40 cycles as probable positive, and ≥ 40 cycles as negative.

### Analyses

**Fig. 1:**
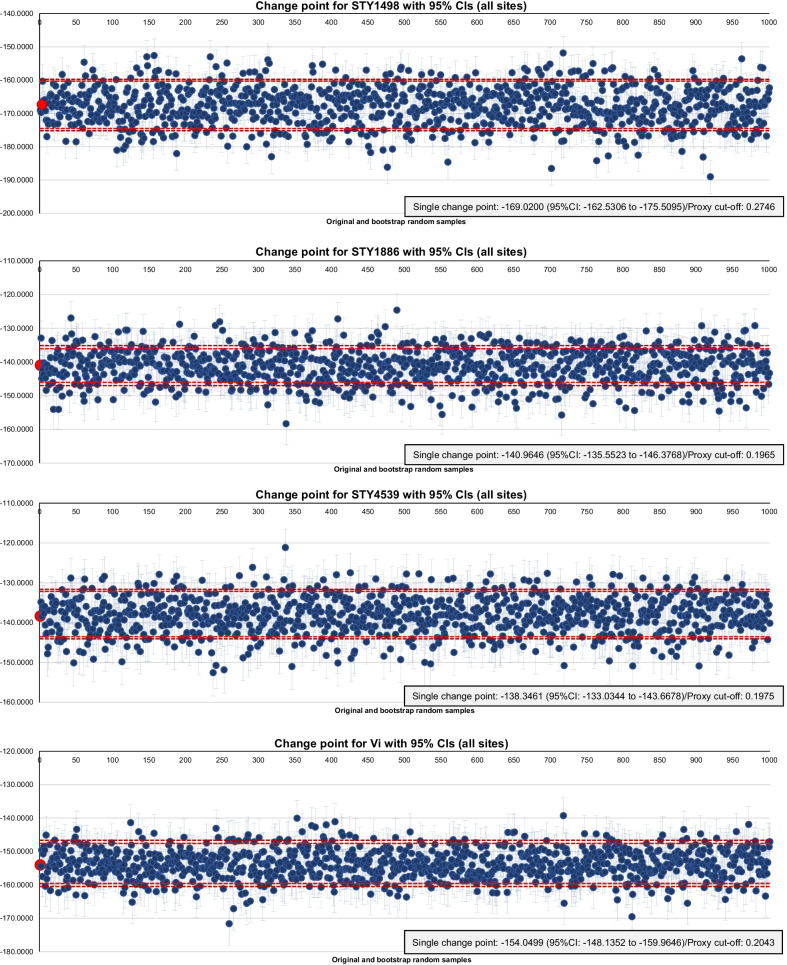
Change point by antigen. Red dot = change point of original data; blue dot = change point of each 1000 bootstrap random samples; light red dashed line = upper and lower 95% confidence interval (CI) of original data; dark red dashed line = upper and lower 99% CI of original data

Participant characteristics were computed as absolute and relative frequencies. *S.* Typhi RT-PCR definitive as well as probable positive and negative were calculated as relative frequencies by site, for both FSs and NFSs. We generated antigen-specific ELISA cut-offs by change-point analyses as initially described by Lardeux et al. [27]. These analyses identify proxy points among ascending ordered absorbance values at points of change in statistical properties or slope of data; this analysis facilitated the separation of negative and positive values in the absence of specific control sera. The cumulative sum (CUSUM) method was used as a detection option coupled with 1000 repetitive bootstrap analyses of original OD values. We applied sampling with replacement for the repetitive analyses. The results presented in Additional file 1: Fig. S2A illustrates overall distributions of original OD values by antigen as absolute and relative cumulative frequencies; Additional file 1: Fig. S3A depicts means, and Fig. 1 presents change points of original and 1000 bootstrap samples accompanied by 95% and 99% confidence intervals (CIs), respectively. We applied antigen-specific proxy points to absorbance values, and calculated absolute positivity and negativity, and prevalence rates (PRs) by site, FSs *versus* NFSs, gender, and age, i.e., < 15 years of age and ≥ 15 years of age. We utilized odds ratios (ORs) with 95%CIs to assess potential correlations between ELISA findings and characteristics congruent among FSs and NFSs inclusive of gender, age, site, and study subjects (Table 2); a two-sided *p-*value of < 0.05 was considered statistically significant. Analyses were performed with STATA statistical package (StataCorp LLC, Version 14, Texas, USA).

**Table 2:** Baseline characteristics of febrile and non-febrile subjects in Ghana, Madagascar and Ethiopia at study enrollment

Country			Ghana			Madagascar					Ethiopia											
Site			Kintampo			Diego			Mahajanga		Addis Ketema W7		Addis Ketema W8		Debre Birhan		Arba Minch		Adama Zuria		Wolayita Sodo	
			Febrile subjects																			
Participants			N = 100			N = 100			N = 100		N = 64		N = 100		N = 104		N = 102		N = 104		N = 100	
Recruitment period [MM/YY]			04/16–08/16			12/15–03/16			01/16–03/16		06/16–10/16		06/16–08/16		06/16–09/16		06/16–09/16		06/16–09/16		06/16–09/16	
Characteristics			N		%	N		%	N	%	N	%	N	%	N	%	N	%	N	%	N	%
Gender		Male	33		33.0	40		40.0	42	42.0	33	51.6	41	41.0	49	47.1	46	45.1	41	39.4	49	49.0
		Female	67		67.0	60		60.0	58	58.0	31	48.8	59	59.0	55	52.9	56	54.9	63	60.6	51	51.0
Age [years]		< 15	53		53.0	40		40.0	61	61.0	0	0.0	1	1.0	7	6.8	3	2.9	5	4.8	8	8.0
		≥ 15	47		47.0	60		60.0	39	39.0	64	100.0	99	99.0	97	93.2	99	97.1	99	95.2	92	92.0
Temperature [°C; axillary]		< 37.5	9		9.0	1		1.0	6	6.0	24	37.5	79	79.0	19	18.3	10	9.8	8	7.7	79	79.0
		≥ 37.5	91		91.0	99		99.0	94	94.0	40	62.5	21	21.0	85	81.7	92	90.2	96	92.3	21	21.0
Recruitment ward		IPD	36		36.0	95		95.0	85	85.0	0	0.0	0	0.0	0	0.0	0	0.0	0	0.0	0	0.0
		OPD	64		64.0	5		5.0	15	15.0	64	100.0	100	100.0	104	100.0	102	100.0	104	100.0	100	100.0
Pre-treatment		Antimalarial	20		20.0	1		1.0	6	6.0	0	0.0	0	0.0	0	0.0	10	9.8	20	19.2	0	0.0
		Antibiotic	9		9.0	18		18.0	70	70.0	2	3.1	0	0.0	2	1.9	1	1.0	40	38.5	0	0.0
Malaria (microscopy/ RDT)		*P. falciparum*	18		18.0	0		0.0	0	0.0	0	0.0	0	0.0	0	0.0	0	0.0	0	0.0	0	0.0
		*P. malariae*	0		0.0	0		0.0	0	0.0	0	0.0	0	0.0	0	0.0	0	0.0	0	0.0	0	0.0
		*P. ovale*	0		0.0	0		0.0	0	0.0	0	0.0	0	0.0	0	0.0	0	0.0	0	0.0	0	0.0
		*P. vivax*	0		0.0	0		0.0	0	0.0	0	0.0	0	0.0	0	0.0	0	0.0	0	0.0	0	0.0
Clinical presumptive diagnosis		ARTI	5		5.0	48		48.0	29	29.0	0	0.0	0	0.0	0	0.0	4	3.9	26	25.0	0	0.0
		Sepsis	13		13.0	0		0.0	23	23.0	0	0.0	0	0.0	0	0.0	0	0.0	4	3.9	0	0.0
		TF	95		95.0	24		24.0	58	58.0	60	93.8	100	100.0	103	99.0	102	100.0	93	89.4	100	100.0
		GTI	9		9.0	49		49.0	55	55.0	1	1.6	0	0.0	0	0.0	0	0.0	21	20.2	0	0.0
		UTI	2		2.0	1		1.0	22	22.0	0	0.0	2	2.0	1	1.0	1	1.0	12	11.5	2	2.0
		CNSI	0		0.0	31		31.0	22	22.0	0	0.0	0	0.0	0	0.0	0	0.0	2	1.9	0	0.0
		Malaria	2		2.0	0		0.0	0	0.0	0	0.0	0	0.0	0	0.0	0	0.0	0	0.0	0	0.0
		FUO	0		0.0	53		53.0	32	32.0	3	4.7	0	0.0	1	1.0	0	0.0	0	0.0	0	0.0
		Other	3		3.0	39		39.0	6	6.0	0	0.0	0	0.0	7	6.7	0	0.0	5	4.8	0	0.0

			Non-febrile subjects																			
Participants			N = 200			N = 200			N = 200		N = 133		N = 199		N = 200		N = 203		N = 196		N = 201	
Recruitment period [MM/YY]			05/16–09/16			12/15–03/16			01/16–03/16		07/16–10/16		06/16–08/16		06/16–10/16		06/16–09/16		06/16–09/16		07/16–09/16	
Characterisitcs			N	%		N	%		N	%	N	%	N	%	N	%	N	%	N	%	N	%
Gender	Male		102	51.0		110	55.0		120	60.0	63	47.4	92	46.2	95	47.5	94	46.3	88	44.9	94	46.8
	Female		98	49.0		90	45.0		80	40.0	70	52.6	107	53.8	105	52.5	109	53.7	108	55.1	107	53.2
Age [years]	< 15		99	49.5		18	9.0		4	2.0	1	0.8	3	1.5	15	7.5	2	1.0	11	5.6	14	7.0
	≥ 15		101	50.5		182	91.0		196	98.0	132	99.2	196	98.5	185	92.5	201	99.0	185	94.4	187	93.0

W7 = Woreda 7; W8 = Woreda 8; IPD = inpatient department; OPD = outpatient department with OR = surgical department and ER = emergency department; *P.* = *Plasmodium*; RDT = rapid diagnostic test; ARTI = acute respiratory tract infection; TF = typhoid fever; GTI = gastrointestinal tract infection; UTI = urinary tract infection; CNSI = central nervous system infection; FUO = fever of unknown origin; antimalarial pre-treatment including range of duration: Ghana: Chloroquine, other (Sulfadoxine/Pyrimethamine), ≥ 1 to ≤ 3 days; Madagascar-Diego: Artemisia, ≤ 3 days; Madagascar-Mahajanga: other (Quinine), ≤ 1 day; Ethiopia-Arba Minch: Artemisia; Ethiopia-Adama Zuria: Chloroquine, Artemisia, ≤ 3 days; Ethiopia-Wolayita Sodo: Chloroquine; antibiotic pre-treatment including range of duration: Ghana: Penicillins (e.g. Penicillin, Cloxacillin, Ampicillin, Amoxicillin, Amoxicillin/Clavulanic acid), oral Cephalosporins (e.g. Cefaclor, Cefpodoxim), Fluoroquinolone (e.g. Ciprofloxacin, Ofloxacin, Moxifloxacin), Metronidazole, ≥ 1 to ≤ 7 days; Madagascar-Diego: Penicillins (e.g. Penicillin, Cloxacillin, Ampicillin, Amoxicillin, Amoxicillin/Clavulanic acid), oral Cephalosporins (e.g. Cefaclor, Cefpodoxim), Macrolide (Erythromycin), Fluoroquinolone (e.g. Ciprofloxacin, Ofloxacin, Moxifloxacin), Metronidazole, Chloramphenicol, Trimethoprim-Sulfamethoxazole (Cotrimoxazole), ≥ 1 to ≤ 7 days; Madagascar-Mahajanga: Penicillins (e.g. Penicillin, Cloxacillin, Ampicillin, Amoxicillin, Amoxicillin/Clavulanic acid), oral Cephalosporins (e.g. Cefaclor, Cefpodoxim), Macrolide (Erythromycin), Fluoroquinolone (e.g. Ciprofloxacin, Ofloxacin, Moxifloxacin), Metronidazole, Chloramphenicol, Trimethoprim-Sulfamethoxazole (Cotrimoxazole), other (Cephalosporin, Gentamycin), ≥ 1 to ≤ 7 days; Ethiopia-Addis Ketema W7: Fluoroquinolone (e.g. Ciprofloxacin, Ofloxacin, Moxifloxacin), ≤ 7 days; Ethiopia-Debre Birhan: Penicillins (e.g. Penicillin, Cloxacillin, Ampicillin, Amoxicillin, Amoxicillin/Clavulanic acid), Fluoroquinolone (e.g. Ciprofloxacin, Ofloxacin, Moxifloxacin), ≥ 2 to ≤ 3 days; Ethiopia-Arba Minch: Fluoroquinolone (e.g. Ciprofloxacin, Ofloxacin, Moxifloxacin), ≤ 2 days; Ethiopia-Adama Zuria: Penicillins (e.g. Penicillin, Cloxacillin, Ampicillin, Amoxicillin, Amoxicillin/Clavulanic acid), Tetracycline (Doxycycline), Fluoroquinolone (e.g. Ciprofloxacin, Ofloxacin, Moxifloxacin), other (Benzylpenicillin), ≥ 7 to ≤ 10 days; Ethiopia-Wolayita Sodo: Penicillins (e.g. Penicillin, Cloxacillin, Ampicillin, Amoxicillin, Amoxicillin/Clavulanic acid), Fluoroquinolone (e.g. Ciprofloxacin, Ofloxacin, Moxifloxacin), ≥ 1 to ≤ 7 days; clinical diagnosis-other: Ghana: gastritis, intestinal parasites, oral ulcers; Madagascar-Diego: viral infection (e.g. arbovirus), parasitic infection (e.g. *Ascaris* spp.), pain/myalgia, gastro-intestinal discomfort, skin infection, rash; Madagascar-Mahajanga: anorexia, congenital central hypoventilation syndrome (CCHS), myalgia, otitis media, hyperthermia; Ethiopia-Debre Birhan: typhus; Ethiopia-Adama Zuria: parasitic infection (e.g. *Schistosoma mansoni*, *Giardia lamblia*, *Entamoeba histolytica*)

## Results

The baseline characteristics of all study participants are summarized in Table 2. Male and female FSs and NFSs were distributed nearly equally across all sites. The FSs and NFSs enrolled in the study were primarily ≥ 15 years of age, except for FSs recruited from the Kintampo and Mahajanga sites. Most FSs had an axillary body temperature of ≥ 37.5 °C, except for those from Addis Ketema Woreda-8 and Wolayita Sodo. Likewise, most FSs were enrolled at out-patient departments as opposed to in-patient departments, except for those from the two Malagasy sites (Diego: 95.0%, 95/100; Mahajanga: 85.0%, 85/100). Antimalarial pre-treatment was reported only rarely by FSs except for Kintampo (20.0%, 20/100) and Adama Zuria (19.2%, 20/104). Soley FSs from both Malagasy sites (Diego: 18.0%, 18/100; Mahajanga: 70.0%, 70/100) and Adama Zuria (38.5%, 40/104) recalled pre-treatment with antibiotics. *Plasmodium falciparum* malaria was detected among FSs recruited in Ghana only. Clinical appraisals revealed typhoid fever as the most common presumptive diagnosis across all sites; other common diagnoses included acute respiratory tract infection in Diego and Adama Zuria, gastrointestinal tract infection at both Malagasy sites, and fever of unknown origin in Diego.

**Fig. 2:**
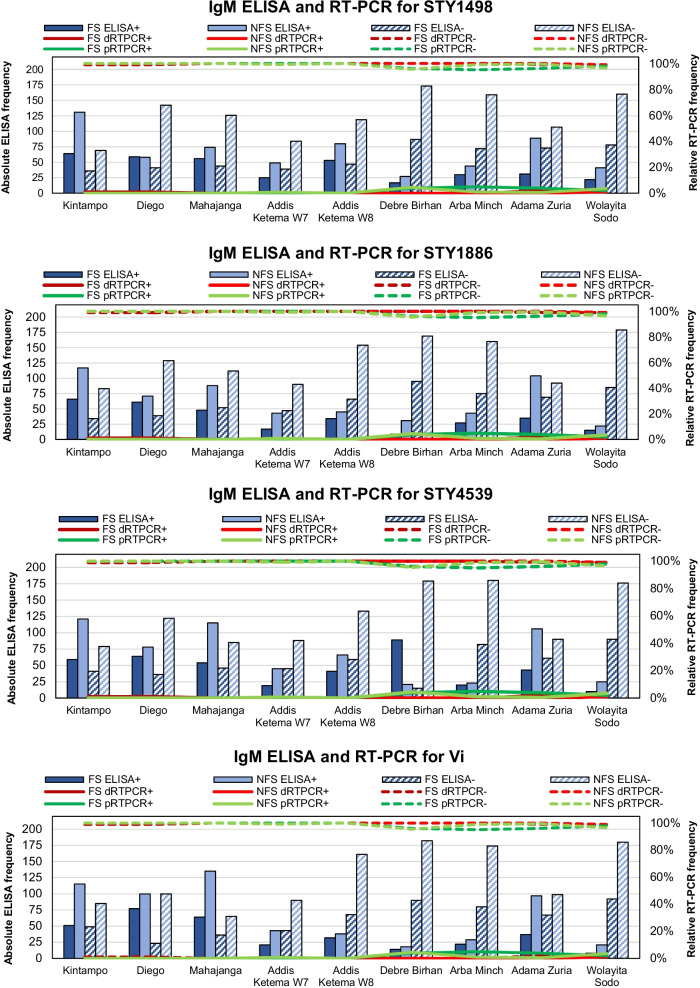
IgM ELISA and RT-PCR by antigen, site and study subjects. W7 = Woreda 7; W8 = Woreda 8; FS = febrile subject; NFS = non-febrile subject; dRTPCR = definitive RT-PCR; pRTPCR = probable RT-PCR; +  = positivity; − = negativity

Definitive positive RT-PCR results were found among FSs in Adama Zuria (1.9%, 2/104), Wolayita Sodo (1.0%, 1/100), Diego (1.0%, 1/100) and Kintampo (1.0%, 1/100), and NFSs in Wolayita Sodo (1.0%, 2/201) (Fig. 2). No *Salmonella* DNA could be amplified from tissue biopsies of two FSs from Arba Minch that underwent surgical procedures for gastrointestinal perforation due to suspected typhoid fever.

**Fig. 3:**
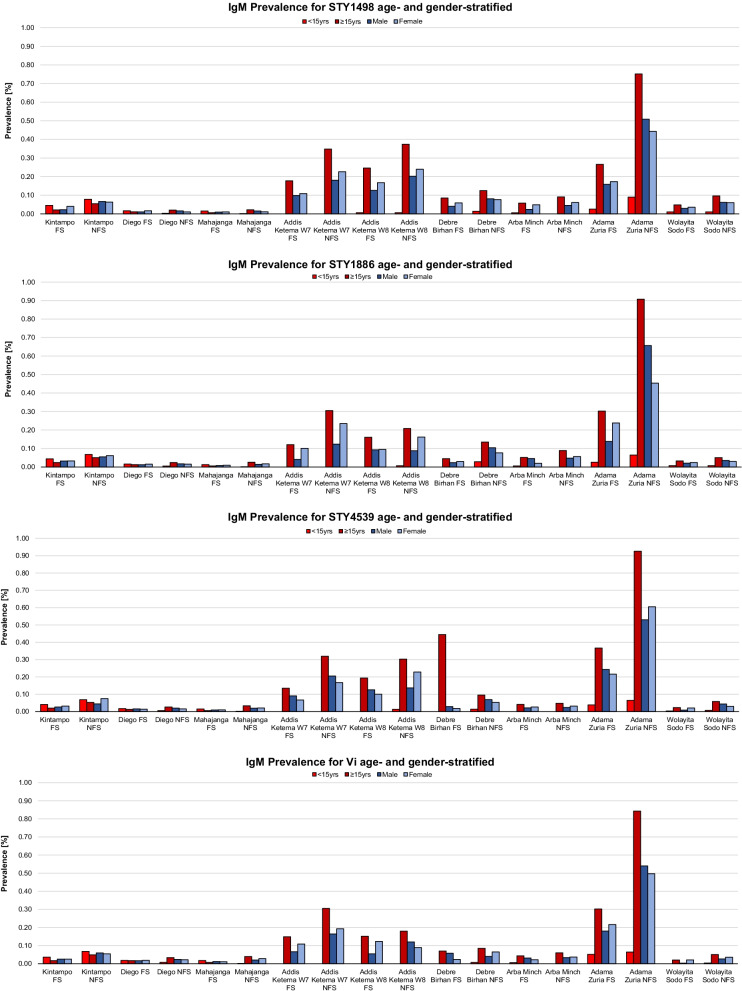
IgM-ELISA by antigen, site and study subjects stratified by age group. W7 = Woreda 7; W8 = Woreda 8; FS = febrile subjects; NFS = non-febrile subjects; yrs = years; + = positivity; − = negativity

Absolute ELISA positivity generated by applying proxy cut-off points (Fig. 1) to absorbance values is shown in Fig. 2. In Kintampo, PRs among FSs (0.05%) and NFSs (0.08%) < 15 years of age were highest for the STY1498 antigen, whereas FSs (0.02%) and NFSs (0.05%) ≥ 15 years of age exhibited strongest responses to STY1886 and STY1498, respectively (Fig. 3). PRs peaked among male FSs as well as male NFSs for STY1886 (0.03%) and STY1498 (0.07%), respectively, and female FSs as well as female NFSs for STY1498 (0.04%) and STY4539 (0.08%), respectively. A 2.45 times (95%CI: 0.98–6.19, *p* = 0.034) higher odds of anti-STY1498 IgM was detected in FSs < 15 years of age compared to FSs ≥ 15 years of age, and a 0.46 times (95%CI: 0.25–0.86, *p* = 0.009) lower odds of anti-STY4539 antibodies in male NFSs than female NFSs.

In Madagascar, positive anti-Vi responses peaked at 0.02% and 0.02% among FSs < 15 and ≥ 15 years of age in Diego, and at 0.02% and 0.01% in Mahajanga, respectively. Both male and female FSs in Diego (0.02% and 0.02%, respectively) and Mahajanga (0.01% and 0.01%, respectively) exhibited the strongest anti-Vi immune responses. In Diego, the odds were 3.21 times (95%CI: 1.24–8.63, *p* = 0.008), 2.31 times (95%CI: 0.91–6.06, *p* = 0.054), and 2.82 times (95%CI: 1.06–7.86, *p* = 0.022) higher for detecting anti-STY1498, anti-STY1886, and anti-STY4539 IgM antibodies in FSs < 15 years of age compared to FSs ≥ 15 years of age, respectively. At the same site, the odds for exhibiting an anti-Vi responses was 2.84 times (95%CI: 0.90–10.55, *p* = 0.048) higher among NFSs < 15 years of age compared to NFSs ≥ 15 years of age.

Across all Ethiopian sites, NFSs exhibited higher IgM responses to all antigens compared to FSs, except for immune responses to STY4539 in FSs recruited at Debre Birhan. FSs and NFSs from Addis Ketema Woreda-7/-8 in Addis Ababa and Adama Zuria presented generally with highest PRs; NFSs from Adama Zuria demonstrated highest PRs, with the highest values detected among male NFSs for STY1498 (0.51%), STY1886 (0.66%), and Vi (0.54%). We also found that the odds were 8.00 times higher (95%CI: 0.74–9.72, *p* = 0.034) for detecting anti-Vi antibodies among FSs in Adama Zuria < 15 year-olds compared to those ≥ 15 years of age. Odds were also 1.96 times higher (95%CI: 1.06–3.62, *p* = 0.020) for detecting a positive response to anti-STY1498 antibodies among male compared to female NFSs.

## Discussion

Following the aim of our investigation, we amplified *S.* Typhi DNA among blood specimens from FSs in Adama Zuria, Wolayita Sodo, Diego and Kintampo, and among blood samples from NFSs in Wolayita Sodo. We also detected IgM-antibodies against all *S.* Typhi antigens investigated among FSs and NFSs across study sites though with site-specific differences; this result is indicative of non-discriminatory stages of acute infections prior to immunoglobulin class-switching due to immunological maturation [23].

HlyE/STY1498 antigen is expressed by several enteric bacteria including *S.* Typhi and *S.* Paratyphi A. It acts as transmembrane pore-forming toxin that induces lysis of erythrocytes and mammalian cells, and promotes apoptosis in macrophages. HlyE/STY1498, therefore, impacts cytotoxicity and intracellular growth within the mononuclear phagocyte system, and is capable of modulating the progression to persistent systemic infections [28–30]. Circulating anti-STY1498 antibodies have been reported in both acute and convalescent/transient cases, although it is unlikely to be detected in carriers as *Salmonella* typically resides in niches that are inaccessible to the host immune system [28, 31]. CdtB/STY1886 is expressed by both, *S.* Typhi and *S.* Paratyphi A, as well as other bacterial pathogens, but rarely in non-typhoidal serovars [28]. CdtB as one of two A-subunits of the AB-type typhoid toxin forms a complex with PltA (pertussis-like toxin A, A-subunit) and PltB (pertussis-like toxin B, B-subunit) that mediates its delivery into host cells [32]. CdtB induces cell cycle arrest via DNA damage that ultimately leads to apoptosis or cell senescence in the short term, as well as long-term consequences that include tumorigenesis, neoplastic lesions, and pathogen persistence [30, 33, 34]. Interestingly, anti-STY1886 immunoglobulins are more likely to develop during convalescence and in response to transient exposure rather than in early acute cases [28, 33]. The PilL/STY4539 is a putative exported protein and a component of the type IV pili fimbrial adhesin that is required for bacterial twitching or gliding motility, adhesion and macrophage-mediated uptake into gut epithelial cells; it also plays an important role in host immune evasion, biofilm formation, and microcolony aggregation [6, 35, 36]. Antibodies to PilL/STY4539 are well detectable among cases suffering from acute infection. Lastly, the Vi carbohydrate capsule is elementary for extracellular survival, protection against neutrophilic oxidative burst, and diminished macrophage responses following ingestion. The antigen appears detectable at a rather late stage of infection, and is presumably associated with *Salmonella* carriage in endemic settings [37]. Protective anti-Vi responses are, based on data obtained through a controlled human challenge model, polyisotypic and polyfunctional [38]. The Vi-antigen enhances the virulence of *S.* Typhi [39] and is an important component of polysaccharide and conjugate vaccine candidates [40]. Studies have shown that anti-Vi immune responses are detectable in individuals from *S.* Typhi-endemic areas; an estimated 19% to 58% of *S.* Typhi exposed individuals have circulating anti-Vi antibodies in their bloodstream [41–43].

Immune responses to all antigens were detected in NFSs across all sites with the strongest PRs identified at the Ethiopian sites. This finding was somewhat surprising as IgM-antibodies have been reported as suitable markers for differentiating acutely infected from healthy individuals [23]. NFSs were presumed to be healthy, i.e., without a history of fever or any clinical indication of a febrile state or infectious pathology within the previous 28 days of recruitment. Among the most likely explanations, the onset in these individuals was asymptomatic or subclinical, and, thus, too marginal to be recognized. It is also possible that previous clinical data were incorrect due to recall and/or reporting bias. Asymptomatic subjects commonly lack fever or signs of infection despite evidence of bacterial species-specific nucleic acid in the bloodstream or fecal shedding [44]. Pathogen-associated factors may promote host survival by favoring limited tissue damage and the establishment of a persistent asymptomatic carrier stage via suppression of initial inflammatory responses [45]. Nevertheless, one weakness of our investigation is that NFSs did not undergo physical examinations and/or clinical appraisals by study physicians to confirm their health status. We also did not cross-check past patient records that may have been available at the recruiting healthcare facilities.

The TSAP and the Global Burden of Disease Study 2017 reported that the highest incidence rates of typhoid fever were detected among school-aged children. Global estimates revealed that 12.6% (range: 8.7–17.7%) of cases and 17.2% (range: 12.3–23.4%) of deaths occurred in children < 5 years of age; furthermore, 55.9% (range: 50.3–61.6%) of cases and 59.3% (range: 53.6–65.2%) of deaths were seen in children < 15 years of age [9–11, 46]. As most of the Ethiopian participants were ≥ 15 years of age in contrast to a more balanced age distribution among Ghanaian and Malagasy subjects, the true prevalence of *S.* Typhi at the Ethiopian sites could have been falsified. Moreover, most of the FSs from Ghana and Madagascar presented with axillary body temperatures of ≥ 37.5 °C and at in-patient and/or out-patient departments, in contrast to FSs that presented solely to out-patient departments at the Ethiopian sites; both are potential indicators of having enrolled subjects with more severe signs of infection. Likewise, participants from Kintampo were recruited at the secondary/tertiary healthcare level compared to the primary/secondary healthcare level at the Malagasy and Ethiopian sites. This indicates a likely different clientele visiting healthcare facilities to seek care. Eligibility criteria of FSs differed by country, which also impacts the direct comparability of our findings. For instance, FSs from Ghana had to have malaria parasites though most enrollees did not fulfill this criterion; FSs from Ethiopia had to have either clinically suspected/diagnosed *Salmonella* infection or febrile illness of unknown reasons. The recruitment period likely did not have any major impact our data as all subjects participated during the country-specific main wet season, i.e. April to July and September to November in Ghana, November to March/April in Madagascar, and June to August/September in Ethiopia [13, 47].

Lardeux et al. [27] did show that change-point analyses are strong in correctly discriminating samples compared to other cut-off equations, which can, thus, minimize chances of misclassification. Caution is advised when classifying absorbance values in close proximity to proxy cut-off points. False results are possible and should be re-confirmed with secondary independent assays. Another limitation of our investigation was that blood specimens were collected once only and in small volumes. This precluded re-testing or bacterial culturing, which is currently considered as the diagnostic gold standard despite varying sensitivity ranging between 45 and 70% [45]. In addition, antibiotic pre-treatment as reported by FSs in Diego, Mahajanga and Adama Zuria is known to impact diagnostic sensitivity though data collected may be incorrect due to recall bias [48]. The bacterial load present in each specimen could have a significant impact on the diagnostic sensitivity of both, antibody detection by ELISA and DNA amplification by RT-PCR [6]. While findings from a *S.* Typhi controlled human challenge model stated detection of bacterial DNA as early as 6–72 h post-exposure, early detection did not predict the likelihood of developing typhoid infection (OR: 0.57, 95%CI: 0.12–2.71, *p* = 0.69) or bacteremia (OR: 0.50, 95%CI: 0.10–2.44, *p* = 0.45) [44].

## Conclusions

In summary, we determined the prevalence of *S.* Typhi using anti-HlyE/-CdtB/-pilL/-Vi-IgM ELISAs and species-specific RT-PCR at sites in Ghana, Madagascar and Ethiopia that have been proposed for implementing surveillance of febrile illness including salmonellosis. Host immune responses revealed the strongest prevalence rates of *S.* Typhi at the Ethiopian sites. This was particulary notable in subjects from the Addis Ababa and Adama Zuria sites, even though the school-aged children, who were presumably at the highest risk, were likely to have been missed. Of note is that literature available for instance in PubMed provides evidence of *Salmonella* in Mahajanga, Adama Zuria, Debre Birhan, Arba Minch and Wolayita Sodo, but not at any of the remaining sites.

When one considers the millions of infections caused by *S.* Typhi each year, together with the impact of recent epidemics due to Ebola virus and COVID-19, the importance of public health surveillance leading to preparedness for immediate responses to disease outbreaks and threats remains of critical importance throughout the African continent. Despite the limitations discussed, our investigation of the utility of both, antigen-specific antibody detection and species-specific nucleic acid amplification, provides useful information that might be used to assess the sites proposed for implementing surveillance of *Salmonella*-associated febrile illnesses as well as other infectious diseases.

## Supplementary Information

**Additional file 1.** Location of sites in Ethiopia [1A], Ghana [1B] and Madagascar [1C]. Notes: The location of each site is indicated as a black dot and the site’s name in red font; Ethiopia (Figure 1A): nine regional states (black italic, capital letters) and two chartered cities (black italic, lower letters) are shown;SNNPR=Southern Nations, Nationalities and People’s Region; Ghana (Figure 1B): ten regions (black italic, capital letters) are shown; Madagascar (Figure 1C): six provinces (black italic, capital letters) are shown.

**Additional file 2.** Distribution of ELISA OD values by antigen.

**Additional file 3.** Mean ELISA ODs by antigen. Notes: OD=optical density; red dot=mean of original data; blue dot=mean of each 1,000 bootstrap random samples; light red dashed line=upper and lower 95% confidence interval (CI) of original data; dark red dashed line=upper and lower 99% confidence interval (CI) of original data

## Data Availability

The datasets used and/or analyzed during the current study are available from the corresponding author on reasonable request. All data generated or analyzed during this study are included in this published article.

## Abbreviations

FSs: Febrile subjects
NFSs: Non-febrile subjects
ELISA: Enzyme-linked immunosorbent assay
RT-PCR: Real-time polymerase chain reaction
OD: Optical density
*S.* Typhi: *Salmonella* Typhi
CUSUM: Cumulative sum
CI: Confidence interval
PR: Prevalence rate
OR: Odds ratio
TSAP: Typhoid Fever Surveillance in Africa Program
SETA: Severe Typhoid Fever in Africa Program
WHO: World Health Organization
INDEPTH: International Network for the Demographic Evaluation of Populations and Their Health
