# Supplementary material for: Detection of Salmonella Typhi nucleic acid by RT-PCR and anti-HlyE, -CdtB, -PilL, and -Vi IgM by ELISA at sites in Ghana, Madagascar and Ethiopia
Source: BMC Infect Dis. 2022 Oct 2;22:766. doi: 10.1186/s12879-022-07726-3 (PMC9526816; doi:10.1186/s12879-022-07726-3)
Supplement: Supplementary file 1 — Additional file 1. Location of sites in Ethiopia [1A], Ghana [1B] and Madagascar [1C]. Notes: The location of each site is indicated as a black dot and the site’s name in red font; Ethiopia (Figure 1A): nine regional states (black italic, capital letters) and two chartered cities (black italic, lower letters) are shown;SNNPR=Southern Nations, Nationalities and People’s Region; Ghana (Figure 1B): ten regions (black italic, capital letters) are shown; Madagascar (Figure 1C): six provinces (black italic, capital letters) are shown. [file 12879_2022_7726_MOESM1_ESM.docx]

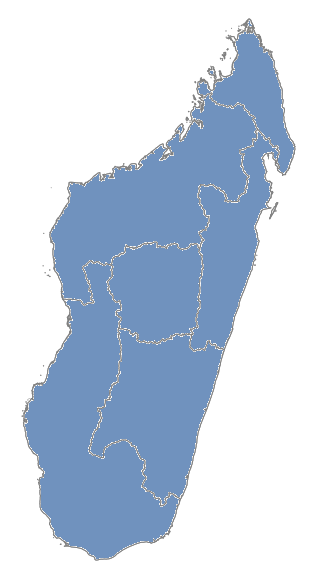


**DIEGO**

**Antsiranana Province**

**KINTAMPO**

**Kintampo District**

**ADDIS ABABA**

**sub-city Ketema Woreda-7**


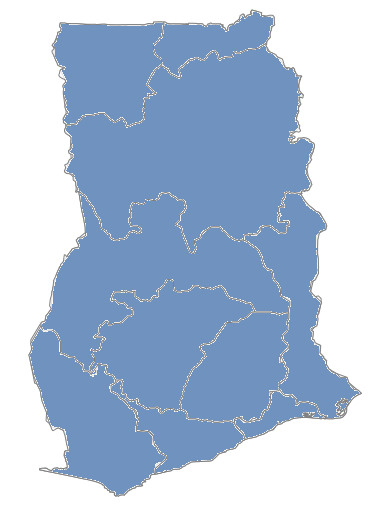
**
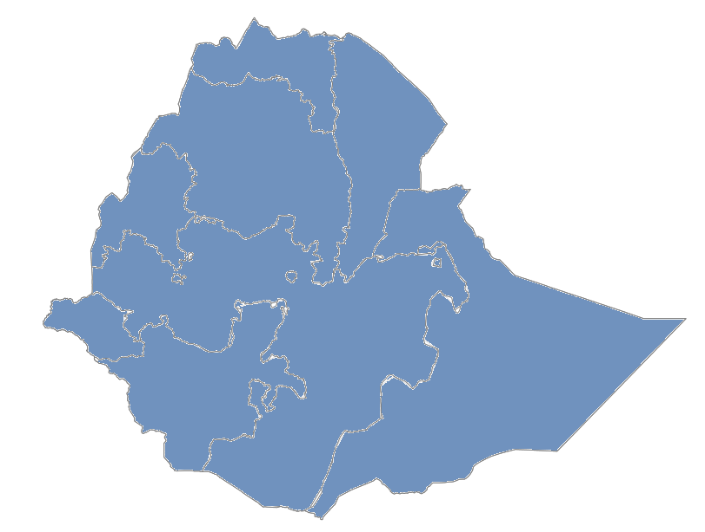
**

**MAHAJANGA**

**Mahajanga Province**

**ADDIS ABABA**

**sub-city Ketema Woreda-8**

*TOLIARA*

*TOAMASINA*

*MAHAJANGA*

*ANTSIRANANA*

*AMHARA*

*BNISHANGUL*

*GUMAZ*

*OROMIA*

*SOMALI*

*UPPER EAST*

*UPPER*

*WEST*

**WOLAYITA SODO Sodo Woreda**

*TIGRAY*

*NORTHERN*

*AFAR*

**DEBRE BIRHAN**

**Debre Birhan Woreda**

*Dire Dawa*

*ANTANANARIVO*

*Addis Ababa*

*BRONG-AHAFO*

*ASHNATI*

*GAMBELA*

*SNNPR*

*VOLTA*

*EASTERN*

*FIANARANTSOA*

*GREATER*

*ACCRA*

*CENTRAL*

*WESTERN*

**[1C]**

**ARBA MINCH**

**Arba Minch Woreda**

**ADAMA WENJI**

**Adama Zuria Woreda**

**[1B]**

**[1A]**

**Additional Figure 1A.** Location of sites in Ethiopia [1A], Ghana [1B] and Madagascar [1C]

Notes: The location of each site is indicated as a black dot and the site’s name in red font; **Ethiopia (Figure 1A):** nine regional states (black italic, capital letters) and two chartered cities (black italic, lower letters) are shown; SNNPR=Southern Nations, Nationalities and People’s Region; **Ghana (Figure 1B):** ten regions (black italic, capital letters) are shown; **Madagascar (Figure 1C):** six provinces (black italic, capital letters) are shown.
