# Supplementary material for: Detection of Salmonella Typhi nucleic acid by RT-PCR and anti-HlyE, -CdtB, -PilL, and -Vi IgM by ELISA at sites in Ghana, Madagascar and Ethiopia
Source: BMC Infect Dis. 2022 Oct 2;22:766. doi: 10.1186/s12879-022-07726-3 (PMC9526816; doi:10.1186/s12879-022-07726-3)
Supplement: Supplementary file 2 — Additional file 2. Distribution of ELISA OD values by antigen. [file 12879_2022_7726_MOESM2_ESM.docx]

**Additional Figure 2A.** Distribution of ELISA OD values by antigen (supplementary)

Note: OD=optical density
