# Supplementary material for: Detection of Salmonella Typhi nucleic acid by RT-PCR and anti-HlyE, -CdtB, -PilL, and -Vi IgM by ELISA at sites in Ghana, Madagascar and Ethiopia
Source: BMC Infect Dis. 2022 Oct 2;22:766. doi: 10.1186/s12879-022-07726-3 (PMC9526816; doi:10.1186/s12879-022-07726-3)
Supplement: Supplementary file 3 — Additional file 3. Mean ELISA ODs by antigen. Notes: OD=optical density; red dot=mean of original data; blue dot=mean of each 1,000 bootstrap random samples; light red dashed line=upper and lower 95% confidence interval (CI) of original data; dark red dashed line=upper and lower 99% confidence interval (CI) of original data [file 12879_2022_7726_MOESM3_ESM.docx]

**Additional Figure 3A.** Mean ELISA ODs by antigen (supplementary)

Notes: OD=optical density; red dot=mean of original data; blue dot=mean of each 1,000 bootstrap random samples; light red dashed line=upper and lower 95% confidence interval (CI) of original data; dark red dashed line=upper and lower 99% confidence interval (CI) of original data
